# Hybridisation between different species of waterfowl

**DOI:** 10.3897/BDJ.14.e197937

**Published:** 2026-08-21

**Authors:** Rusko Petrov, Slavko Nikolov

**Affiliations:** 1 Department of General animal husbandry, Faculty of Veterinary Medicine, Trakia University, Stara Zagora, Bulgaria Department of General animal husbandry, Faculty of Veterinary Medicine, Trakia University Stara Zagora Bulgaria; 2 Department of Anatomy, physiology and animal sciences, Faculty of Veterinary Medicine, University of Forestry, Sofia, Bulgaria Department of Anatomy, physiology and animal sciences, Faculty of Veterinary Medicine, University of Forestry Sofia Bulgaria; 3 Institute of Neurobiology, Bulgarian Academy of Sciences, Sofia, Bulgaria Institute of Neurobiology, Bulgarian Academy of Sciences Sofia Bulgaria

**Keywords:** Anatinae, captive breeding, crossbreeding, duck, heredity, hybrid, inheritance, phenotypic variation, teal

## Abstract

When waterfowl are kept in mixed collections in captivity, interbreeding between different species can often be observed. Five cases of hybridisation between different ducks and teals raised together have been described. The first case from 2011 was a cross between a male Hottentot teal (*Spatula hottentota*) and a female Silver teal (*Spatula versicolor*), from the same genus Spatula, the offspring resembling the mother in size, while in decoration, it primarily took on the features of the father. In a second hybridisation from 2018, between a male Ferruginous duck (*Aythya nyroca*) and a female Mandarin duck (*Aix galericulata*), the hybrids strongly resembled the father. In 2020, the third hybridisation occurred between the male Brazilian teal (*Amazonetta brasiliensis*) and the female Ringed teal (*Callonetta leucophrys*), with their offspring more similar in colour and size to the father. In 2025, two cases of interspecific hybridisation were observed between a male Baikal teal (*Sibirionetta formosa*) and a female Falcated duck (*Mareca falcata*), with the offspring having predominantly dominant paternal traits. The latest case of hybridisation is between a Chestnut teal (*Anas castanea*) and a Wood duck (*Aix sponsa*), with the offspring being intermediate in traits between the species.

## Introduction

Interspecific hybridisation amongst waterfowl is a well-documented biological phenomenon that has garnered increasing scientific attention in recent years, particularly regarding ex situ breeding and conservation efforts (Muñoz‐Fuentes et al. 2007, Holopainen et al. 2015, Huang et al. 2025). In the wild, interspecific crossing in waterfowl occurs relatively infrequently due to established ecological, behavioural and geographical barriers (Tubaro and Lijtmaer 2002, Taysom 2016, Chen et al. 2023). However, when maintained in mixed-species captive collections, these reproductive barriers are significantly diminished, thereby facilitating the occurrence of hybridisation (Guay et al. 2014, Gu et al. 2023, Huang et al. 2025).

Species of ducks and teals are characterised by close phylogenetic relationships, which frequently facilitate the production of viable offspring (Mank et al. 2004, Ouassou et al. 2021, Kumari et al. 2026). This study examines small-sized wild waterfowl species of the genus *Spatula*, Hottentot teal and the Silver teal, alongside South American teals, specifically the Brazilian teal and the Ringed teal. Hottentot teals, *Spatula hottentota* (Eyton, 1838), exhibit pronounced sexual dimorphism; the species is characterised by a bright blue bill, a dark brown crown and a cream-coloured face contrasted by dark-coloured cheeks, along with a distinctively speckled brown body (Koopman 2021). Females construct small nests on the ground, typically laying clutches of six to nine eggs (Breed et al. 2023). Their habitat range encompasses eastern and southern Africa (Breed et al. 2020). In contrast to the aforementioned species, Silver teals *Spatula versicolor* (Vieillot, 1816) inhabit Central to southern America (Desch 2023). They are distinguished by a dark black crown, white cheeks and a bicoloured bill that is bright blue at the base with yellow-green hues (Oyarzún-Ruiz et al. 2024). Their nest most commonly consists of a shallow depression in the soil, where the female lays between six and ten eggs (van Els et al. 2024).

Brazilian teals, *Amazonetta brasiliensis* (Gmelin, 1789), are a South American species inhabiting the Amazon River Basin (Silva et al. 2020). The species is distinguished by its light brown body, striking blue-green and white wing patches that are highly visible during flight and deep red legs; notably, only the male possesses a bright red bill (Studer and Crozariol 2022). The nest is a shallow cup constructed from interwoven wet plant fibres, reeds and leaves, with a clutch size ranging from eight to fourteen eggs (Campos et al. 2025). The Ringed teal, *Callonetta leucophrys (*Vieillot, 1816), is an exceptionally small and striking duck exhibiting pronounced sexual dimorphism (Bulgarella et al. 2010). Males feature a chestnut back, a pinkish breast speckled with black spots, grey flanks and abdomen and a characteristic black stripe encircling the head (Provini et al. 2012). In contrast, the female is dark brown with pale eyebrows and facial striping (Silva et al. 2020). Both sexes possess a blue-grey bill and large white wing patches that contrast with a green speculum during flight (Montani et al. 2019). These teals utilise natural tree cavities and hollows, where the female lays and incubates a clutch of six to twelve white eggs (Gereg 2012).

Additionally, species such as the Ferruginous duck, Mandarin duck, Wood duck, Baikal teal and Falcated duck are included. Ferruginous ducks, *Aythya nyroca* (Güldenstädt, 1770), occur in Europe; they are medium-sized waterfowl adapted for efficient submergence and diving underwater (Djelailia et al. 2017). The plumage is predominantly chestnut-brown with a white abdomen (Fouzari et al. 2015). The most distinctive feature of adult males is the striking white colouration of the iris (Houhamdi and Samraoui 2008). Females construct nests from emergent and riparian vegetation on the ground near the water or the shoreline, laying between seven and eleven eggs which they incubate alone (Loucif et al. 2021). The Mandarin duck, *Aix galericulata* (Linnaeus, 1758), is also a medium-sized duck inhabiting the forested regions of East Asia, renowned as one of the most beautiful and colourful birds on the planet (Ng et al. 2022). Breeding males are easily recognisable, featuring a red bill, a large white crest above the eyes, a reddish face and characteristic orange "sails" (fan-like feathers) that project vertically from the back (Bellebaum and Mädlow 2015). The female is much more cryptic, displaying a grey-brown plumage with pale spots on the flanks and a white eye-ring (YiJin et al. 2019). Mandarin ducks are typical cavity-nesting waterfowl, breeding in tree hollows high above the ground, ranging from 6 - 15 metres (Hu 2025). On average, the female lays nine to twelve cream-coloured eggs, which she incubates independently (Sun et al. 2014).

The Chestnut teal, *Anas castanea (*Eyton, 1838), is a small waterfowl species endemic to Australia (Dhami et al. 2012). The species exhibits pronounced sexual dimorphism; males possess a glossy, iridescent green head, a rich chestnut-coloured breast and abdomen and a dark back (Joseph et al. 2009). The female features a cryptic colouration, characterised by an entirely mottled brown body pattern (Lindsey and Roderick 2011). This species displays plasticity in its nesting site selection, breeding both in tree cavities and on the ground in shallow depressions (Kennedy and Spencer 2000). Typically, the female lays a clutch of seven to ten eggs (Guay 2011). Wood ducks, *Aix sponsa* (Linnaeus, 1758), are highly adapted to an arboreal lifestyle and are distributed throughout North America (Dyson et al. 2018). Males exhibit strikingly beautiful plumage, characterised by an iridescent green-and-purple crest, white stripes on the neck, a chestnut breast speckled with white dots and bright red eyes and bills (Davis et al. 2010). The female is much more cryptic — grey-brown with a distinctive white eye-ring, providing excellent camouflage in the wild (Davis et al. 2009). The wood duck is an obligate cavity nester, utilising holes and crevices (Kennamer et al. 2005). It constructs its nest in natural tree hollows situated 1 - 15 metres above the ground, where females lay clutches of 10 to 16 eggs (Hepp and Kennamer 2012).

The Baikal teal, *Sibirionetta formosa* (Georgi, 1775), is a small, yet exceptionally striking waterfowl species (Zelenkov et al. 2023). They inhabit the taiga zones of Eastern Siberia and the region surrounding Lake Baikal, from which their name originates (Nam et al. 2025). During the breeding season, the male's head is adorned with a complex geometric pattern of green, yellow (cream) and black patches, separated by narrow white lines (Sakda et al. 2023). The female exhibits a cryptic brown colouration, distinguished by a characteristic, well-defined white spot at the base of the bill and a dark stripe passing through the eye (Manglani et al. 2023). Additionally, the male features elongated and elegant scapular feathers that drape down over the wings (Lenkiewicz and Stawarczyk 2022). The nest consists of a shallow depression on the ground, where the female lays a clutch of six to nine eggs (Tajir et al. 2015). The Falcated duck, *Mareca falcata* (Georgi, 1775), is a large-bodied dabbling duck species native to East Asia (Zhang et al. 2020). Breeding males possess a large, spectacular head with iridescent metallic green and bronze colouration, complemented by a long crest draping down towards the nape (Abhinav and Dhadwal 2017). Their most remarkable feature is the series of elongated, narrow and sickle-shaped inner secondary feathers (tercials) that hang elegantly over the tail (Duck 2005). The female displays a cryptic brown plumage typical of most female ducks, but is distinguished by a longer, entirely black bill and grey legs (Carboneras and Kirwan 2020). For nesting, the female utilises a shallow scrape on the ground, laying a clutch of seven to ten eggs (Meng et al. 2019).

These waterfowl are distinguished by diverse morphological and behavioural traits, including specific plumage colouration, courtship rituals and adaptations to various ecological niches, rendering them particularly compelling subjects for investigating interspecific hybridisation (Liu and Churchil 2022, Guillemain et al. 2025, Gess et al. 2026). Such crossbreeding provides opportunities to trace inheritance patterns and phenotypic expression in hybrid progeny (Dhami et al. 2015, Lavretsky et al. 2019, Amponsah et al. 2019). These processes serve as a valuable source of data regarding genetic compatibility, hereditary mechanisms and the manifestation of morphological traits (Cole and Wood 2017, Liu and Churchil 2022, Zhang et al. 2024). Concurrently, hybridisation raises critical questions concerning the preservation of genetic integrity, particularly when managing rare and endangered populations (Khirani-Betrouche and Moulaï 2022, Nugmanova et al. 2024).

Despite an increasing number of observations, detailed descriptions of specific hybridisation events remain limited, particularly within southeast Europe. To address this gap, the present study documents and analyses several instances of interspecific hybridisation amongst waterfowl maintained in captivity, with a primary emphasis on evaluating the morphological characteristics, reproductive performance and inheritance patterns of the resulting hybrid offspring.

## Material and methods

The present study was conducted on waterfowl maintained in mixed-species captive ornamental collections, where various representatives of the subfamily Anatinae co-habitated in communal aviaries. The observation period spanned from 2011 to 2025, during which a total of five independent hybridisation events were documented, encompassing both interspecific and intrageneric crosses.

The first documented case of hybridisation dates back to 2011, involving a cross between a male Hottentot teal and a female Silver teal; however, the exact clutch size was not determined. In 2018, a hybridisation event was recorded between a male Ferruginous duck and a female Mandarin duck, with a total of nine eggs laid. In 2020, a cross was observed between a male Brazilian teal and a female Ringed teal, resulting in a clutch of six eggs. Two separate hybridisation events were documented in 2025: one involving a male Chestnut teal and a female Wood duck and another between a male Baikal teal and a female Falcated duck. In both 2025 cases, the precise number of eggs laid remains unknown, as they were collected and incubated under ex situ conditions.

The first three cases were identified in a mixed collection comprising 16 to 24 waterfowl species over the years, located in the village of Voyvodinovo, Plovdiv Province, Bulgaria. The aviary measures 12 m × 6 m × 4 m (height), with a water surface area of 3.5 m × 2.5 m and a depth of 0.5 m. Nesting boxes were provided for cavity-nesting species, positioned at various heights ranging from 0.5 m to 2–2.5 m above the ground (to accommodate pinioned, non-flying and full-winged flying individuals). Additionally, shallow wooden trays were placed on the ground for open-nesting species.

The second facility, where the fourth hybridisation event was recorded, is located in the village of Pancharevo, Sofia Province, Bulgaria. This collection numbers approximately 30–35 species of visually diverse ducks, teals, swans and diving ducks. The aviary measures 25 m × 13 m × 6 m, with an even sex ratio maintained amongst the birds. Multiple nesting shelters are available, including natural hollow logs, rock formations and artificially installed nesting boxes for wood ducks. The pond measures 10 m × 6 m with a depth of 1 m. The aviary is equipped with large branches and perches positioned at varying heights above the ground.

The third waterfowl collection is situated in the village of Chintulovo, Sliven Province, Bulgaria, where the fifth hybridisation event was documented. The collection consists of approximately 26–28 waterfowl species with an even sex ratio. The aviary measures 20 m × 14 m × 8 m and features abundant natural vegetation. It includes a natural water body measuring 9 m × 7 m with a depth of 0.6 m. Nesting boxes are provided both at elevated positions and on the ground, alongside vegetated areas suitable for ground-nesting bird species.

The animals under study were maintained under standardised conditions within enclosed environments providing access to waterbodies, natural vegetation and opportunities for the expression of species-typical behaviour, including spontaneous pair formation. Nutrition was provided through balanced compound feeds, grain mixtures and supplementary natural food sources, tailored to the specific dietary requirements of each species.

Hybrid pair formation occurred spontaneously, without the application of deliberate experimental protocols for controlled crossbreeding. Partner selection took place within social groups, allowing for the analysis of natural mechanisms of sexual selection and interspecific interactions in waterfowl.

Data collection for each case included detailed records of the species affiliation and sex of the parental individuals, clutch size, hatching success and offspring survival to sexual and morphological maturity, alongside descriptions of the primary phenotypic characteristics of the hybrids. In specific instances, eggs were collected for artificial incubation, a factor accounted for in the subsequent analysis of the results.

The development of the hybrid offspring was monitored through systematic visual surveillance, photographic documentation and longitudinal tracking of individuals. The evaluation of morphological and phenotypic traits was based on a comparative analysis relative to the parental forms, examining indicators such as plumage colouration, body dimensions, physical proportions, behavioural responses and the presence of sexual dimorphism.

The resulting data were analysed using a descriptive-analytical approach aimed at identifying patterns in the inheritance of phenotypic traits, as well as the degree of dominance and variability of parental characteristics across different hybrid combinations. Particular emphasis was placed on the capacity for producing viable offspring and the manifestation of intermediate or dominant phenotypic features in the hybrid individuals.

English common names and taxonomic treatment follow The Howard and Moore Complete Checklist of the Birds of the World, 4^th^ edition (Dickinson and Remsen 2013). Taxonomic authority citations are formatted in accordance with standard zoological nomenclature.

## Results

Under captive conditions, where waterfowl are maintained in mixed-species collections, both interspecific and intrageneric hybridisation occur, facilitated by the absence of natural geographical and behavioural barriers. Within the scope of the present study, a total of five hybridisation events between various duck and teal species were documented. Of these, four represented intergeneric crosses (involving the genera *Aythya*, *Aix*, *Amazonetta*, *Callonetta*, *Anas*, *Mareca* and *Sibirionetta*), while one instance was identified as an intrageneric cross (genus *Spatula*).

The first instance of hybridisation was identified in 2011, involving an intrageneric cross between a male Hottentot teal (*Spatula hottentota*) and a female Silver teal (*Spatula versicolor*). The exact clutch size was not determined due to the practice of egg collection and artificial incubation. From the resulting progeny, only one male offspring reached advanced developmental stages (Fig. 1). The observed hybrid exhibits phenotypic affinity to the maternal species regarding body size, being significantly larger, consistent with the Silver teal. Conversely, the plumage colouration more closely resembles the paternal phenotype, strongly favouring the Hottentot teal (Fig. 1a). The bill lacks the yellow basal spot and the white plumage on the rump is absent, both of which are characteristic of the maternal silver teal phenotype (Fig. 1b). However, the hybrid differs from the paternal Hottentot teal phenotype by the absence of the black triangular patch within the white facial area and the black crown is notably darker.

In 2018, a hybridisation event was recorded between a male Ferruginous duck (*Aythya nyroca*) and a female Mandarin duck (*Aix galericulata*). This combination resulted in a clutch of nine eggs, from which three individuals hatched. Two offspring survived to maturity: one male (Fig. 2) and one female (Fig. 3). The resulting hybrids exhibit a pronounced phenotypic resemblance to the paternal species, with a clear dominance of its morphological characteristics. Both sexes possess a more elongated body structure, characteristic of the Mandarin duck, rather than the typically robust, rounded form of the Ferruginous duck. Regarding plumage, they inherit the phenotypic expression of the father. The male hybrid features a light iris (Fig. 2a), a sexual trait consistent with the Ferruginous duck, though it displays a slight darkening of the head and grey feathers on the flanks, which are atypical for the paternal species (Fig. 2b). The female hybrid predominantly mirrors the paternal colouration, with the distinction (Fig. 3a) that the white patch under the bill and the white triangles on the wing speculum are absent (Fig. 3b).

The third instance, identified in 2020, involves a cross between a male Brazilian teal (*Amazonetta brasiliensis*) and a female Ringed teal (*Callonetta leucophrys*). A clutch of six eggs was laid between 16 June and 24 June 2020, with hatching recorded on 20 July 2020. Although six ducklings hatched, three perished at the age of two days, while the remaining three individuals reached maturity. All surviving hybrids are male and exhibit predominantly paternal phenotypic characteristics regarding size and colouration (Fig. 4). The primary body and head colouration strongly resemble the paternal phenotype of the Brazilian teal. Influence from the maternal Ringed teal genome is evident in the bill colouration, which is blue in two of the hybrids (Fig. 4a) and black in the third (Fig. 4b). Furthermore, the legs are pink, whereas in typical male Brazilian teals, they should be a vivid scarlet red.

Two separate hybridisation events were documented in 2025. The first involved a cross between a male Chestnut teal (*Anas castanea*) and a female Wood duck (*Aix sponsa*). The exact clutch size was not determined due to the practice of egg collection and artificial incubation. This cross resulted in two male hybrids exhibiting a mixed phenotype with intermediate characteristics, although the paternal phenotype appears dominant (Fig. 5). The heads of both hybrids are dark-coloured, a trait shared by both parental species; however, the distinctive white facial markings characteristic of the Wood duck are absent (Fig. 5a). The dorsal region is dark, while the abdomen and breast are creamy-brown, a colouration consistent with both parental species. The bill structure resembles that of the Wood duck, but it is significantly darker, reflecting the paternal influence of the Chestnut teal (Fig. 5b). Additionally, two female hybrids (Fig. 6) were identified, which phenotypically closely resemble the paternal species; the head and body exhibit a plumage pattern similar to that of the chestnut teal (Fig. 6a). Conversely, the morphology of the bills and the presence of a pale patch beneath the bill are characteristic features of female wood ducks (Fig. 6b).

The final case from 2025 involves a cross between a male Baikal teal (*Sibirionetta formosa*) and a female Falcated duck (*Mareca falcata*). The exact clutch size remains unknown, as the eggs were collected and incubated under ex situ conditions. Two individuals hatched from this progeny — one male (Fig. 7) and one female (Fig. 8) — both demonstrating phenotypic traits characteristic of both parental species, consistent with an intermediate inheritance pattern. The male hybrid strongly resembles the paternal Baikal teal, with the exception that the facial markings and colour zones are not clearly differentiated (Fig. 7a); a blending of colours occurs from the breast towards the flanks, lacking the distinct white vertical line characteristic of the paternal species (Fig. 7b). The female hybrid phenotypically inherits more traits from the male parent, yet lacks the white spot at the base of the bill (Fig. 8a) and exhibits a larger body size, consistent with the Falcated duck (Fig. 8b).

The synthesised results demonstrate that successful mating occurred in all observed cases of captive hybridisation, with viable offspring produced in the majority of instances. Phenotypic analysis reveals that paternal traits predominated in most hybrids, though intermediate inheritance was also noted in specific pairings. These findings support the hypothesis that, when natural reproductive barriers are absent, waterfowl are capable of producing hybrids with significant phenotypic and genetic variability.

## Discussion

Important evolutionary and conservation issues are being revealed. The artificial overcoming of behavioural and ecological barriers in captivity facilitates interspecific hybridisation within the order Anatidae, underscoring the risk of genetic swamping and introgression into wild populations should such individuals escape into the wild (Johnsgard 1960, Díez-del-Molino et al. 2020). From a conservation perspective, the uncontrolled crossing of ducks in captive collections diverts valuable resources away from scientifically managed ex-situ conservation programmes aimed at preserving genetically pure and threatened taxa (Čížková et al. 2012, Loucif et al. 2021).

As key traits such as plumage structure and colouration in ducks are often determined by complex polygenic interactions and epistasis, relying solely on morphological assessment limits the accurate determination of parental contribution and inheritance patterns (Mank et al. 2004, Huang et al. 2025). The external resemblance of some offspring to a single parental species can mask cryptic hybridisation, which necessitates that future studies combine phenotypic analysis with molecular markers to validate parental dominance (Muñoz‐Fuentes et al. 2007, Chen et al. 2023).

Observations indicate that captive environments not only increase the frequency of interspecific interactions, but also facilitate successful reproduction between species that would rarely, if ever, encounter one another in the wild (Stephens et al. 2019, Rohwer et al. 2022, Kumari et al. 2026). In several of the described cases, viable offspring were produced, though variations were observed in survival rates, hatching success and the attainment of sexual maturity (Ahmad et al. 2024, Cabrera and Zarco 2024). This further underscores the importance of controlled environments in the study of hybridisation processes.

The findings of the present study confirm that captive waterfowl environments provide favourable conditions for both interspecific and intrageneric hybridisation. The absence of natural geographical barriers, coupled with the opportunity for spontaneous pair formation within mixed-species collections, leads to an increased probability of crossbreeding — a phenomenon rarely observed in the wild (Trivedi and Trivedi 2019, Stephens 2020, Ottenburghs and Harteman 2021).

The documented cases demonstrate that hybridisation can occur successfully even between representatives of different genera, suggesting significant genetic compatibility within the subfamily Anatinae (Galimberti et al. 2018, Joseph 2018, de Souza et al. 2019). The observed variations in clutch size, hatchability and offspring survival rates indicate that reproductive success in such crosses is inconsistent and depends on the specific combination of parental species (Mank et al. 2004, Muñoz‐Fuentes et al. 2007, Bonczek and Ringelman 2021).

The analysis of the phenotypic characteristics of the resulting hybrids reveals distinct variations in the degree of inheritance of parental traits. In a portion of the cases, a dominance of paternal characteristics is observed, particularly regarding colouration and overall morphological appearance, whereas in other combinations, the offspring demonstrate an intermediate phenotype, blending traits from both parental species (Tubaro and Lijtmaer 2002, Nielsen and Lehmhus 2022). This is consistent with established inheritance models in birds, where genetic expression can be influenced by several factors, including sex-linked inheritance and genetic imprinting (Licuanan et al. 2017, Wang et al. 2018, Chen et al. 2023).

Of particular interest is the observation that, in most documented cases, paternal characteristics exhibit a more pronounced phenotypic expression (Gu et al. 2023, Huang et al. 2025). This phenomenon may be attributed to specificities in genetic regulation or to asymmetry in the inheritance of certain traits, including those related to colouration and sexual dimorphism (Amponsah et al. 2019, Nugmanova et al. 2024, Lavery 2024). Such observations are consistent with existing literature, which indicates that waterfowl hybrids frequently inherit dominant traits from one parent, often the male (Williams 2017, Zhou et al. 2018, Gess et al. 2026).

From a conservation and evolutionary perspective, the hybridisation events documented in this study provide valuable insights into the permeability of reproductive barriers amongst waterfowl species maintained under captive conditions. Although hybridisation can contribute to our understanding of species relationships, inheritance patterns and mechanisms of gene flow, it may also present challenges for ex situ conservation programmes. Uncontrolled hybridisation in captive collections could potentially compromise the genetic integrity of pure populations, particularly when threatened or conservation-dependent species are involved. Therefore, careful management of breeding groups remains essential to balance scientific research objectives with long-term conservation goals.

Conversely, the variability in offspring survival rates underscores that hybridisation does not consistently result in the formation of viable populations (Lavretsky et al. 2019, Liu and Churchil 2022). The observed losses during the early stages of development in certain cases may be attributed to genetic incompatibility or the reduced adaptability of hybrid individuals (Quilodrán et al. 2018, Aguillon et al. 2025). This indicates that, while hybridisation is possible, it does not always yield a sustainable generation (Mengting et al. 2019, Ouassou et al. 2021).

It should also be acknowledged that the interpretation of inheritance patterns in the present study is based primarily on phenotypic observations. While plumage colouration, body morphology and external characteristics provide important indicators of parental influence, phenotypic assessment alone may not fully reflect the underlying genetic architecture of hybrid individuals. The apparent predominance of paternal traits observed in several hybrid combinations should, therefore, be interpreted with caution, as trait expression may also be influenced by sex-linked inheritance, epigenetic mechanisms, incomplete dominance or environmental factors. Future studies incorporating molecular and genomic analyses would help clarify patterns of inheritance and provide a more comprehensive understanding of parental contributions to hybrid offspring.

The findings of this study are significant for understanding hybridisation processes in waterfowl under conditions of artificial co-habitation and emphasise the role of environmental factors in overcoming natural reproductive barriers. The resulting data contribute to a better understanding of the genetic compatibility between diverse species and the mechanisms that dictate phenotypic expression in hybrid individuals.

## Conclusion and conservation significance

The findings of this study demonstrate that captive environments create a significant potential for both interspecific and intrageneric hybridisation by removing natural geographical and behavioural barriers. The documented cases confirm that diverse species within the subfamily Anatinae can produce viable offspring, with phenotypic expression ranging from intermediate blends to a clear dominance of paternal traits. These results underscore the complexity of genetic interactions and the influence of hereditary mechanisms in the development of hybrid waterfowl.

These findings carry significant implications for both scientific research and conservation management. While hybridisation in natural ecosystems is typically restricted by spatial isolation, behavioural differences and distinct ecological adaptations, captive environments remove these barriers. This can lead to the emergence of hybrid lineages that may, in some cases, suffer from reduced viability or altered adaptability. Consequently, the careful management of mixed-species collections is essential to prevent unwanted crossbreeding, particularly when dealing with rare or endangered species.

From a conservation perspective, monitoring reproduction and the co-habitation of diverse species in captivity is essential for preserving the genetic integrity of populations. The occurrence of hybridisation under such conditions can lead to genetic pollution, posing a significant risk in the event of potential re-introduction or the utilisation of individuals in conservation programmes.

Concurrently, the study of hybridisation provides valuable insights into genetic compatibility between diverse species and can serve as a model for understanding evolutionary processes and inheritance mechanisms. This holds significance for both theoretical biology and practical applications in breeding activities and the management of populations under controlled conditions.

In conclusion, the present study underscores the necessity of a balanced approach between scientific inquiry and conservation objectives, with an emphasis on preserving species diversity and preventing uncontrolled hybridisation in waterfowl.

## Figures and Tables

**Figure 1a.**
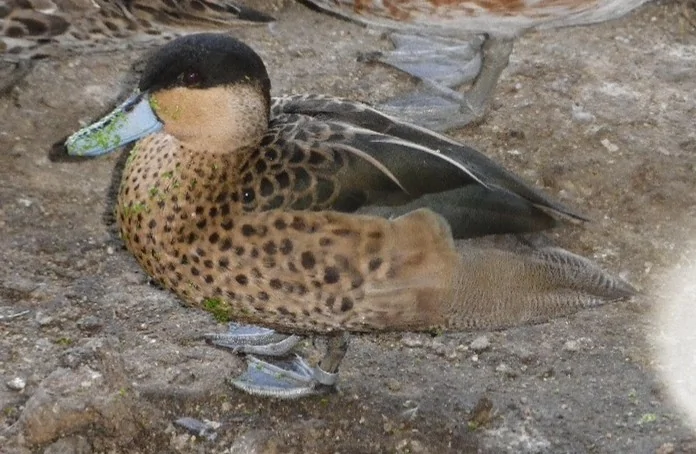
Left lateral view;

**Figure 1b.**
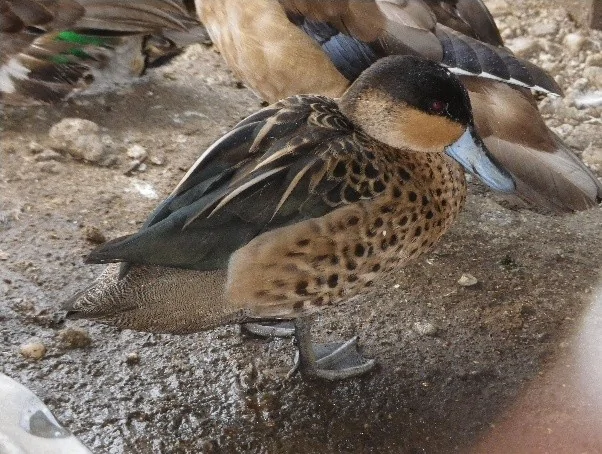
Right lateral view.

**Figure 2a.**
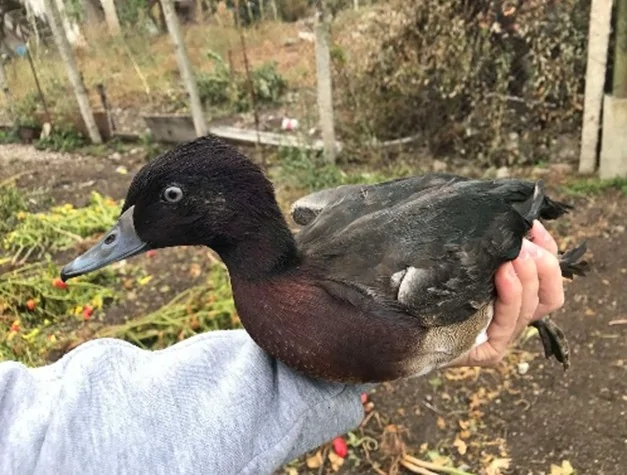
Left lateral view;

**Figure 2b.**
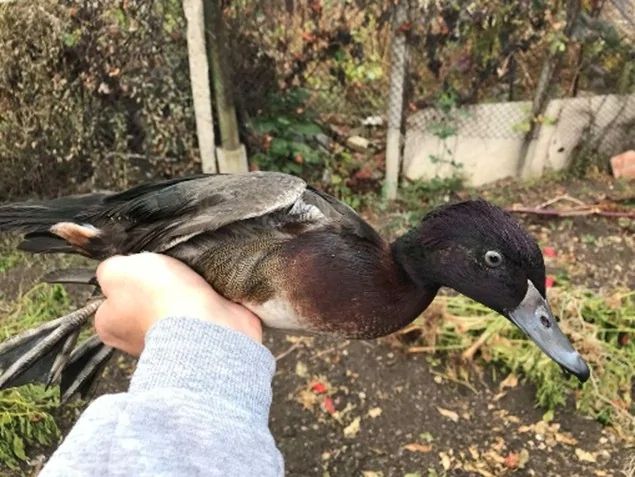
Right lateral view.

**Figure 3a.**
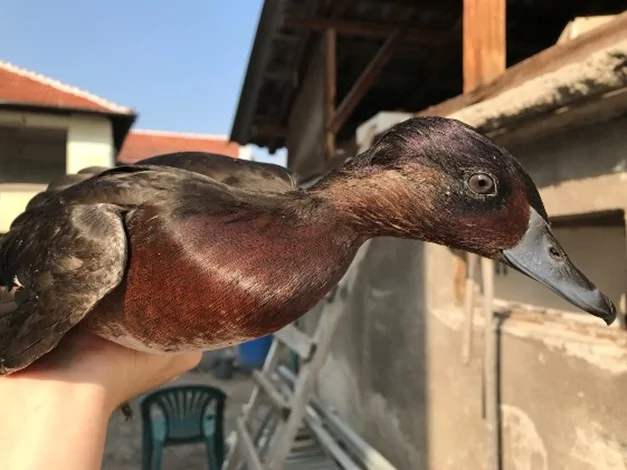
Right lateral view, close-up;

**Figure 3b.**
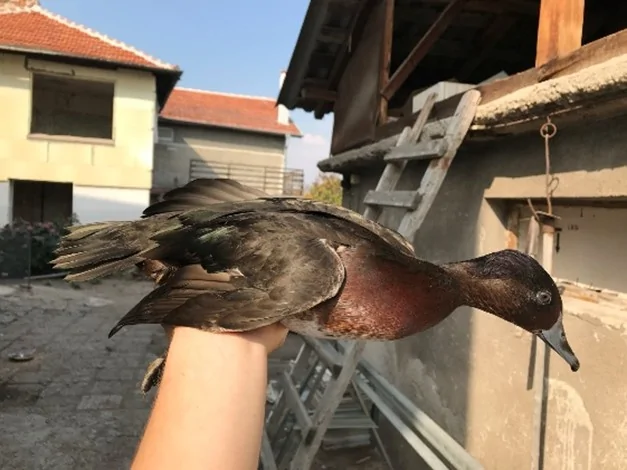
Wide-angle view.

**Figure 4a.**
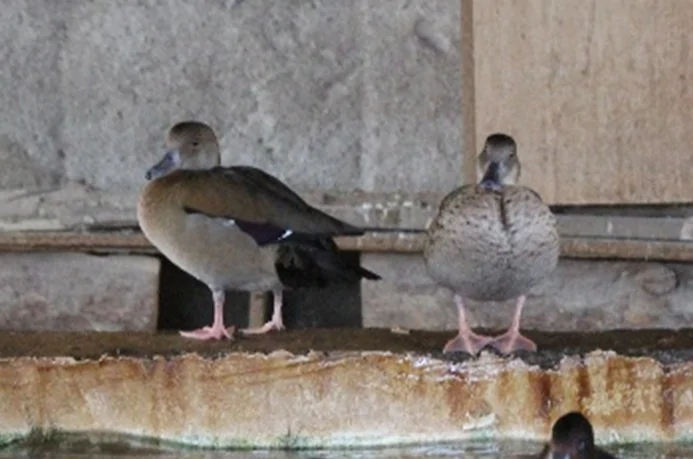
Profile and frontal views;

**Figure 4b.**
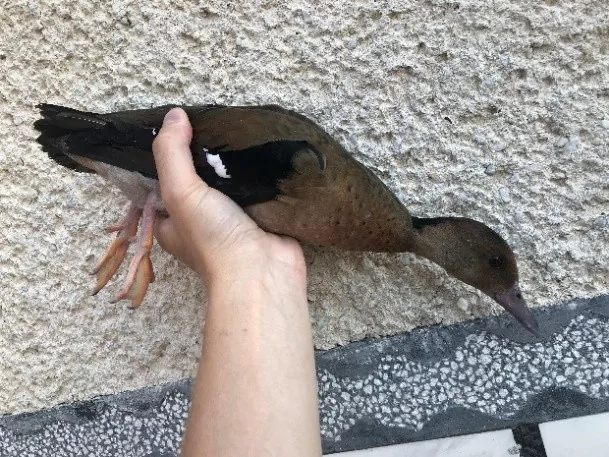
Right lateral view.

**Figure 5a.**
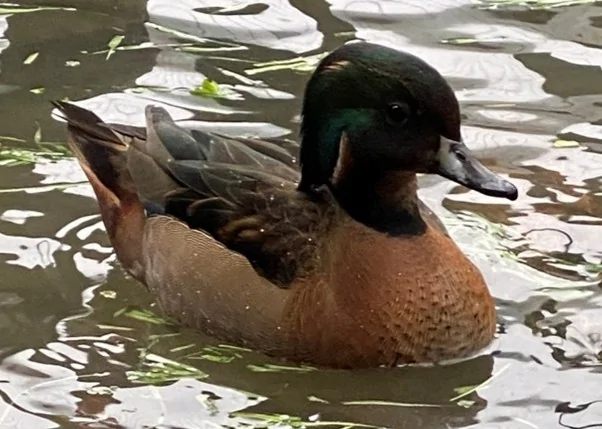
Right lateral view of the first;

**Figure 5b.**
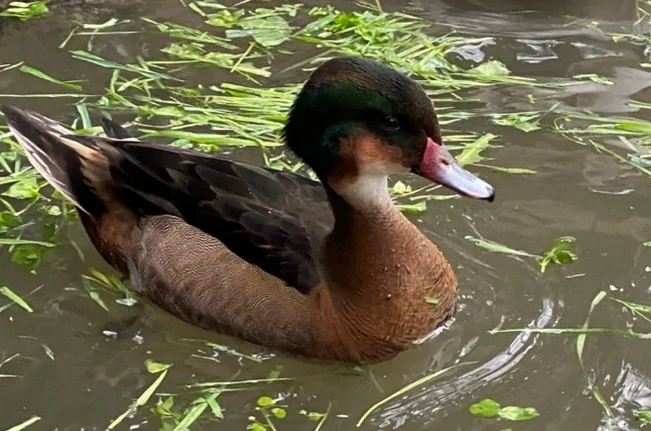
Right lateral view of the second.

**Figure 6a.**
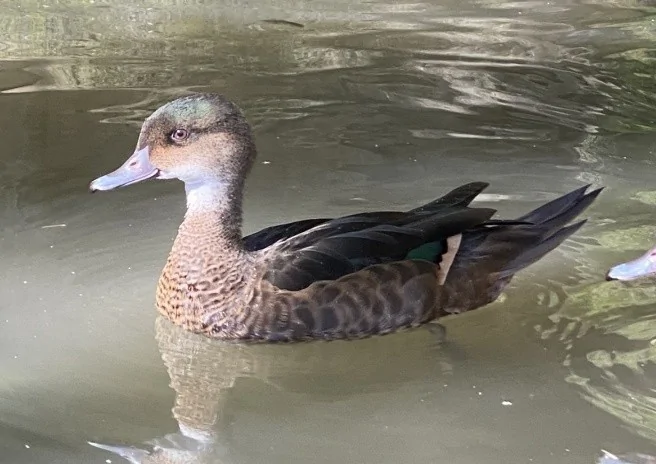
Right lateral view of the first female hybrid;

**Figure 6b.**
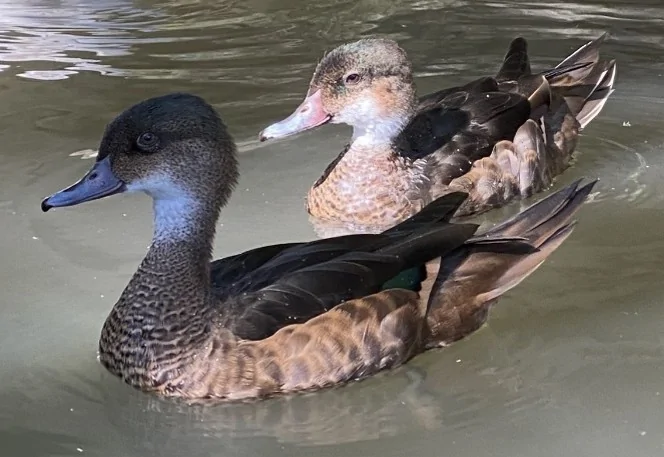
Right lateral view of the second female hybrid.

**Figure 7a.**
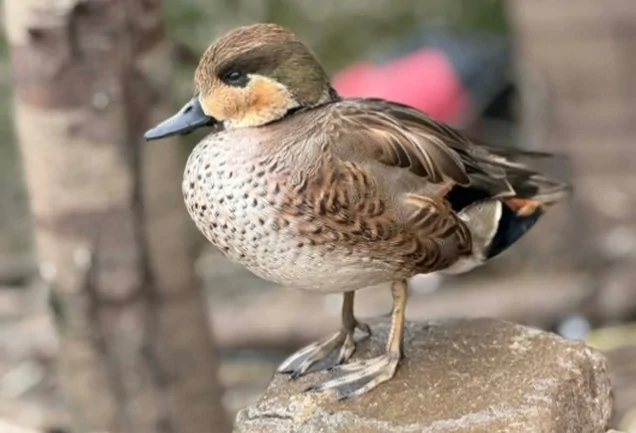
Left lateral view;

**Figure 7b.**
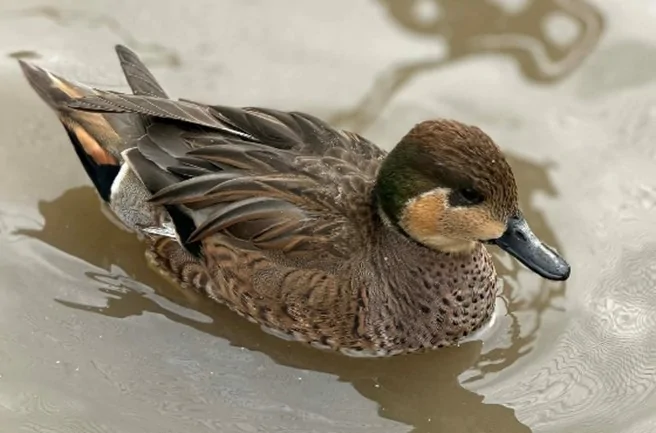
Right lateral view.

**Figure 8a.**
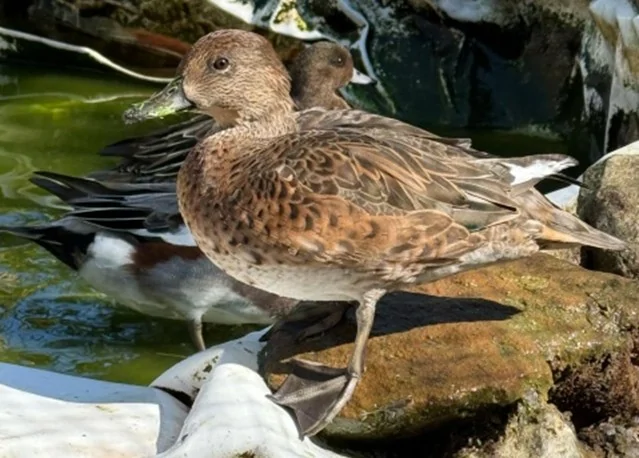
Left lateral view;

**Figure 8b.**
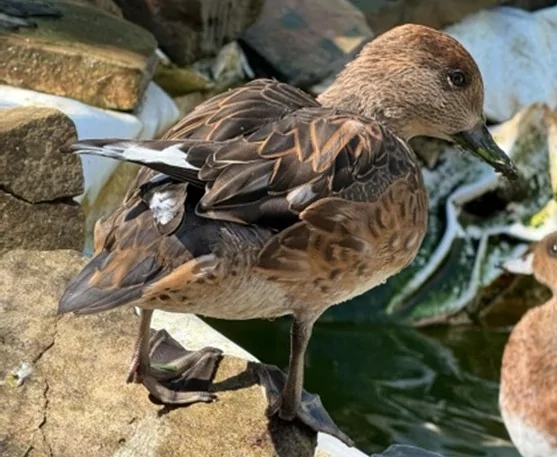
Right lateral view.
